# Aspirin plus tirofiban inhibit the thrombosis induced by Russell’s viper venom

**DOI:** 10.1186/s12959-016-0093-1

**Published:** 2016-10-04

**Authors:** Ren-Chieh Wu, Ping-Tse Chou, Li-Kuang Chen

**Affiliations:** 1 Department of Emergency Medicine, Tzu Chi Medical Center, Hualien, Taiwan; 2 Department of Laboratory Diagnosis, School of Medicine, Tzu Chi University, Hualien, Taiwan; 3 Branch of Clinical Pathology, Department of Laboratory Medicine, Tzu Chi Medical Center, Hualien, Taiwan

**Keywords:** Russell’s viper venom, Mouse model, Thrombosis, Antiplatelet

## Abstract

**Background:**

Thrombosis and coagulopathy are the commonest hematological manifestations of envenomation of Russell’s viper venom (RVV). Factor X is activated by a factor X-activating enzyme from Russell’s viper venom (RVV-X) to start the coagulation cascade. We established an animal model with local ischemic effects induced by RVV. We tried to treat RVV envenomation with antiplatelets and anticoagulants without recourse to antivenom.

**Methods:**

RVV was injected into the foot pad of mice. We observed the effects at different intervals and compared local changes in ischemia with drug treatment after 30 min.

**Results:**

A combination of aspirin plus tirofiban could prevent the ischemic change induced by RVV. The antithrombotic effects of single-use of aspirin or tirofiban were better than single-use of heparin or clopidogrel.

**Conclusion:**

The aspirin + tirofiban group had a better outcome with respect to prevention of tissue ischemia and gangrene. This indicates that the activation and aggregation of platelets is the major cause of thrombosis induced by RVV.

## Background

*Daboia russelli* (“Russell’s viper”) is distributed throughout ten South-East Asian countries, including Taiwan [1]. The effects of Russell’s viper venom (RVV) can lead to many different severe conditions such as coagulopathy, thrombotic microangiopathy [2], stroke [3], renal failure [4], generalized increase in capillary permeability, and rhabdomyolysis and neurotoxicity. However, these effects can vary among the different subspecies [5, 6]. Incoagulable blood caused by consumption coagulopathy (including disseminated intravascular coagulation (DIC) and thrombocytopenia) is one of the commonest features and the leading cause of death due to RVV across the entire geographical distribution of the species [5–7]. DIC induced by RVV can lead to massive occlusion of the renal microvasculature with fibrin deposition and parenchymal ischemia [8] and may be a predisposing factor of acute renal failure. Disseminated thrombus formation has been shown to develop in the large vessels of small animals bitten by Russell’s viper [7, 9]. DIC with coagulation factors activated by an activator of factor X from the RVV can eventually lead to the production of stabilized fibrin, which could be the reason for vessel obstruction [7]. Systemic thrombosis was reported in 15 % of patients with systemic envenoming from *Daboia russelli formosensis* (Formosan Russell’s viper) [10]. From our experience, the severity of DIC, renal failure, and thrombocytopenia caused by Formosan Russell’s viper venom is associated with clinical outcome (Wu et al. unpublished data). We wanted to create an animal model for the hemorrhagic property of Formosan Russell’s viper venom. Anticoagulation agents or antiplatelet agents could then be tested to see if they could prevent venom-induced thrombosis and sub sequent organ damage.

Thrombosis occludes vessels, which then leads to local tissue ischemia; the subsequent tissue necrosis is suspected to be the leading cause of multiple-organ damage. An animal model of RVV-induced thrombosis has not been reported. We constructed an animal model with measurement of local changes in cyanosis, gangrene, mummification, and tissue necrosis after injection of a sub-lethal dose of RVV into the foot pad of mice to mimic the thrombosis caused by RVV. We then used the model to test aspirin, clopidogrel, tirofiban and heparin for the prevention of venom-induced vessel occlusion and tissue necrosis.

## Methods

### Materials

All snakes were obtained in eastern Taiwan. The venom of *Daboia russelli formosensis* was collected directly from the snakebite through parafilm in a test-tube every month. Each batch of venom was pooled from a one-year collection of more than eight Formosan Russell’s vipers. We tested the LD50 of RVV in 25-gmice via the intraperitoneal route.

### Animal model

Female NMRI mice from the National Animal Center (age, 6–8 weeks; 25 ± 3 g) were used. Aspirin, tirofiban, clopidogrel and heparin were the anticoagulant drugs used.

Prior to anticoagulant agent injections, sub-lethal doses of RVV (0.05 μL) were injected into the left foot pads of each experimental mice. This dosage was chosen because the LD_50_ through this inject route was demonstrated in literature, in tested mice, to be 0.1 μL. Subsequently, anticoagulant agents were injected via the intraperitoneal route 30 min after envenomation. The degree of local ischemic change and the change in kinetics at different time intervals were compared with the drugs treated 30 min after envenomation.

### Drugs

Aspirin (brand name, Stin; manufactured by China Chemical & Pharmaceutical Company Limited, Taiwan), tirofiban (Aggrastat; MSD, USA), clopidogrel (Plavix; Sanofi Aventis, France) and heparin (Agglutex; China Chemical & Pharmaceutical) were used.

### Effects of aspirin

Eighteen mice were divided into three groups of six. All mice were injected with 0.05μLvenom in the left foot pad. Group 1 was treated once with aspirin (10 mg/kg, i.p.). Group 2 was treated with aspirin once (40 mg/kg, i.p.). Group 3 was the control group and had no treatment. The observation time intervals were day-1, day-2, and day-7.

### Effects of tirofiban plus another drug

Thirty mice were divided into five groups of six mice were injected with 0.05μLvenom into their left foot pad. Except for the control group, all other mice were injected with tirofiban (12 mg/kg) initially and every 8 h until the experiment was complete. Group 1 was the control group and agents were not administered. Mice in group 2 were injected with tirofiban only. Group 3 contained mice that were additionally injected once with aspirin (10 mg/kg, i.p.). Group 4 contained mice that were additionally injected once with clopidogrel (1 mg, i.p.). Group 5 contained mice that were additionally injected with heparin (5 units, i.p.). The observation time interval was 16 h and 48 h after injection.

### Effects of clopidogrel and heparin

In addition to the control group, two groups of six mice were also studied. Group 1 was administered clopidogrel (1 mg/25 g weight, i.p.) and group 2 was given heparin (5 units, i.p.). Changes were observed at 8, 16, and 48 h.

## Results

### The thrombogenic mouse foot model

**Fig. 1:**
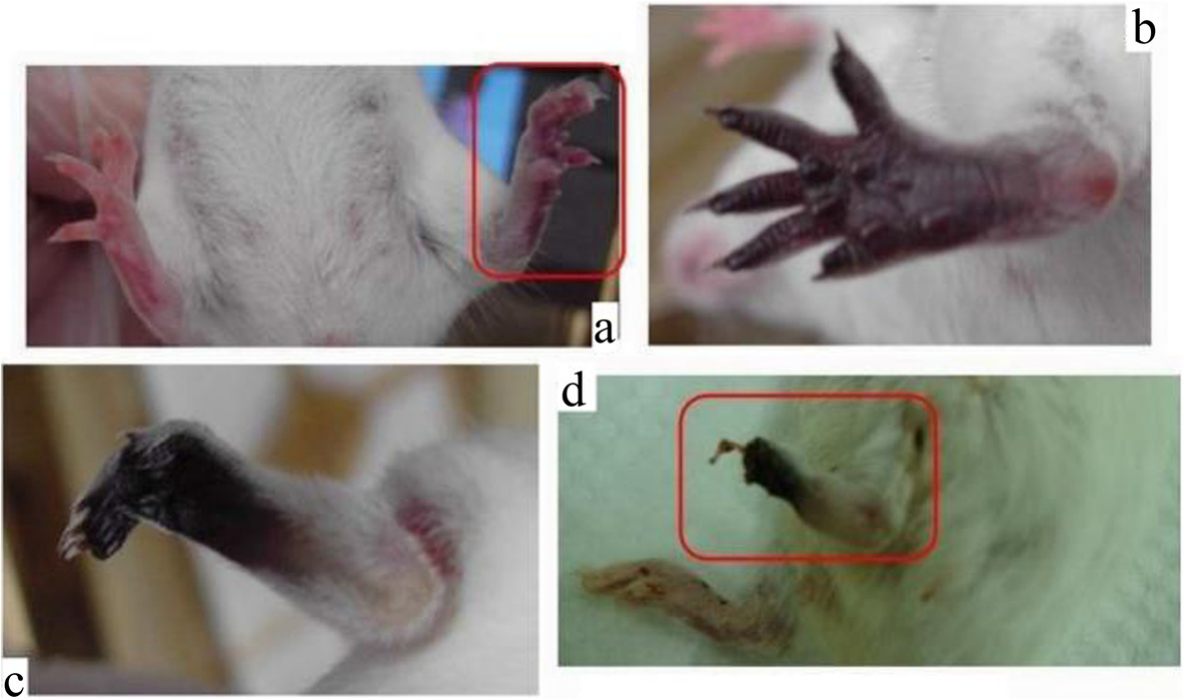
The natural course of venom, injected at foot of mice. **a** venom injected 15 min later. **b** venom injected 1 day later. **c** venom injected 2 days later. **d** venom injected 7 days later

The degree and kinetics of ischemia in local tissue was constant among the six mice in each group (Tables 1 and 2). The natural course of envenoming in mice feet is shown in Fig. 1. We observed changes in mice feet at 15 min, 16 h, 48 h and 7 days after envenoming. Fifteen minutes after envenoming, local tissue cyanosis was noted. A gangrenous change in mice feet was observed 16 h after envenoming. Mummification of mice feet was observed after 48 h. Foot necrosis was observed on day7 (Fig. 1). The distinction of mummification (dry gangrene) and gangrene was made because gangrene, when properly treated, is reversible. However tissue mummification is irreversible and will ultimately lead to tissue necrosis.

**Table 1:** Preventative effects of antiplatelets and anticoagulants against tissue ischemia induced by RVV

Treatment	Percentage of ischemic change (%)			
	Cyanosis	Gangrene	Mummification	Necrosis
Negative (venom only)	100 % (6/6)	100 % (6/6)	100 % (6/6)	100 % (6/6)
Aspirin (As) low	100 % (6/6)	100 % (6/6)	100 % (6/6)	100 % (6/6)
Aspirin high	100 % (6/6)	100 % (6/6)	0 % (0/6)	0 % (0/6)
Tirofiban(T)	100 % (6/6)	100 % (6/6)	0 % (0/6)	0 % (0/6)
Heparin (H)	100 % (6/6)	100 % (6/6)	100 % (6/6)	100 % (6/6)
Clopidogrel (C)	100 % (6/6)	100 % (6/6)	100 % (6/6)	100 % (6/6)
Ag and C	100 % (6/6)	100 % (6/6)	0 % (0/6)	0 % (0/6)
Ag and H	100 % (6/6)	100 % (6/6)	0 % (0/6)	0 % (0/6)
Ag and As	0 % (0/6)	0 % (0/6)	0 % (0/6)	0 % (0/6)

**Table 2:** Effects and distribution of tissue damage induced by RVV envenomation reacted with treatment of antiplatelets and anticoagulants

Treatment	Degree of ischemic change			
	Cyanosis	Gangrene	Mummification	Necrosis
Negative (venom only)^a^	All plantar	All plantar	All plantar	All plantar
Aspirin (As) low	All plantar	All plantar	3/4 plantar	1/2 plantar
Aspirin high	All plantar	1/2 plantar	none	none
Tirofiban (T)^b^	Half plantar	Digits	none	none
Heparin (H)	All plantar	1/2 plantar	1/2 plantar	1/2 plantar
Clopidogrel (C)	All plantar	1/2 plantar	1/2 plantar	1/2 plantar
Ag and C^c^	All plantar	3/4 plantar	none	none
Ag and H^c^	Digits	Digits	none	none
Ag and As^b^	none	none	none	none

^a^Reference to Fig. 1

^b^Reference to Fig. 4

^c^Reference to Fig. 5

### Effects of aspirin at different doses

**Fig. 2:**
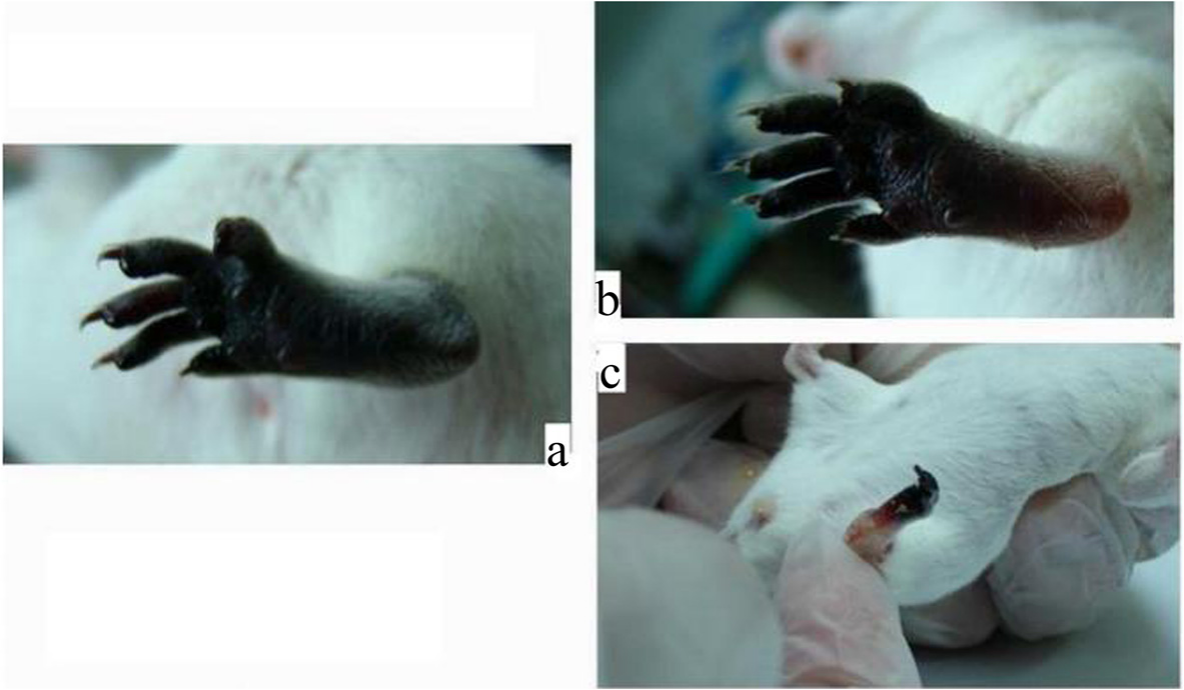
The effects of low dose (10 mg/kg) post-envenomation Aspirin treatment at day 1, 2 and 7. **a** 1 day later. **b** 2 days later. **c** 7 days later

**Fig. 3:**
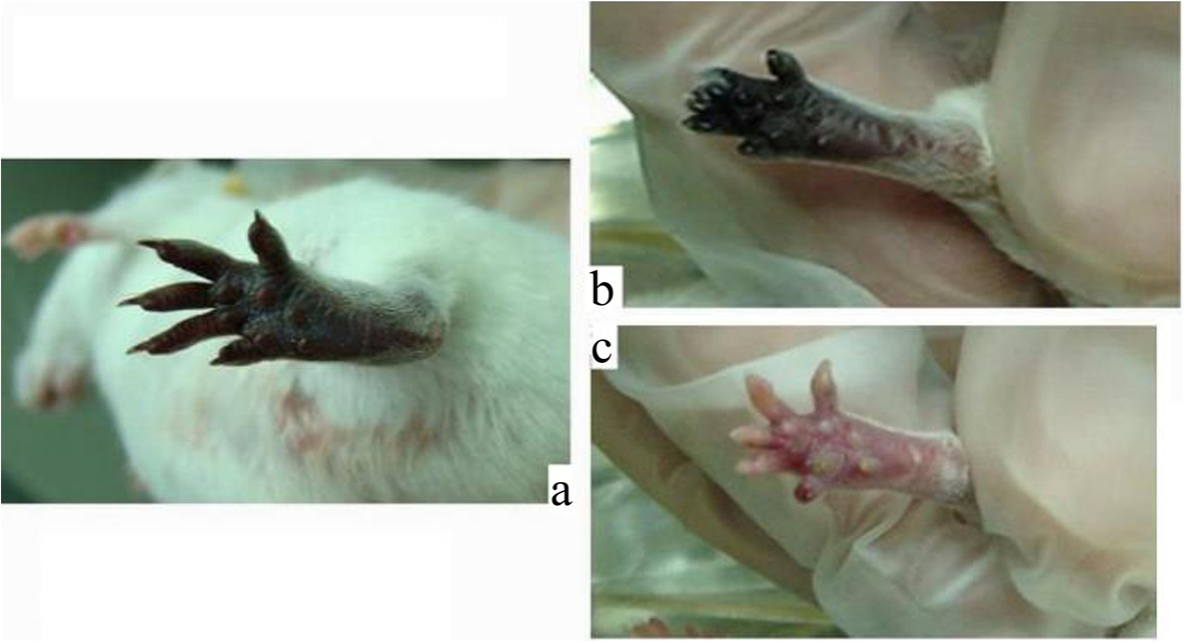
The effects high dose (40 mg/kg) post-envenomation Aspirin treatment at day 1, 2 and 7. **a** 1 day later. **b** 2 days later. **c** 7 days later

A high dose of aspirin (40 mg/kg) was better than a lower dose (10 mg/kg) (Figs. 2 and 3). Aspirin showed no obvious effects on early ischemic change in mice feet, but prevented subsequent progression to gangrene (Fig. 3). The lower-dose aspirin group showed gangrenous change 24 h after envenomation, and mummification at day7. The higher-dose aspirin group showed ischemic changes and gangrene at 24 h and 48 h; the condition of foot recovered at day7, but local swelling persisted. Overall, all mice did not completely recover, but outcome in the treated group was better than that in the control group at identical observation points.

### Combined use of tirofiban with aspirin/clopidogrel/heparin

**Fig. 4:**
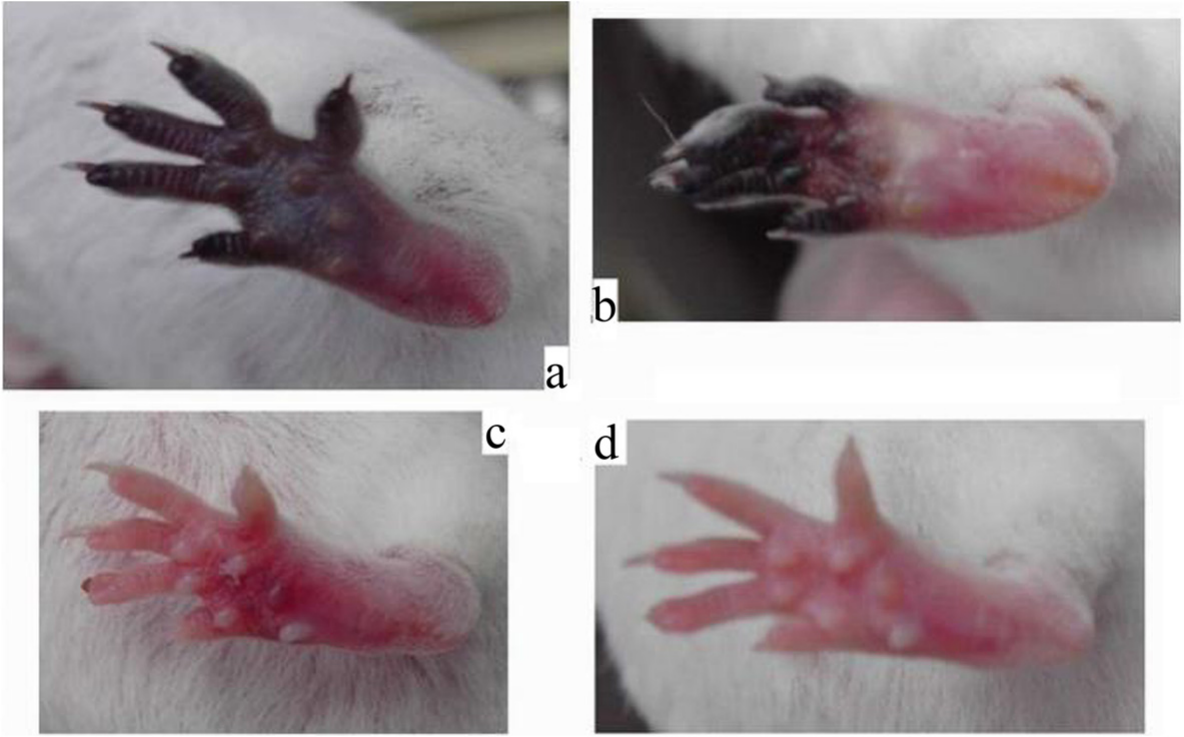
The effects of post-envenomation treatment with tirofiban and aspirin. **a** One day after tirofiban treatment. **b** Two days after tirofiban treatment. **c** One day after tirofiban + aspirin treatment. **d** Two days after tirofiban + aspirin treatment

**Fig. 5:**
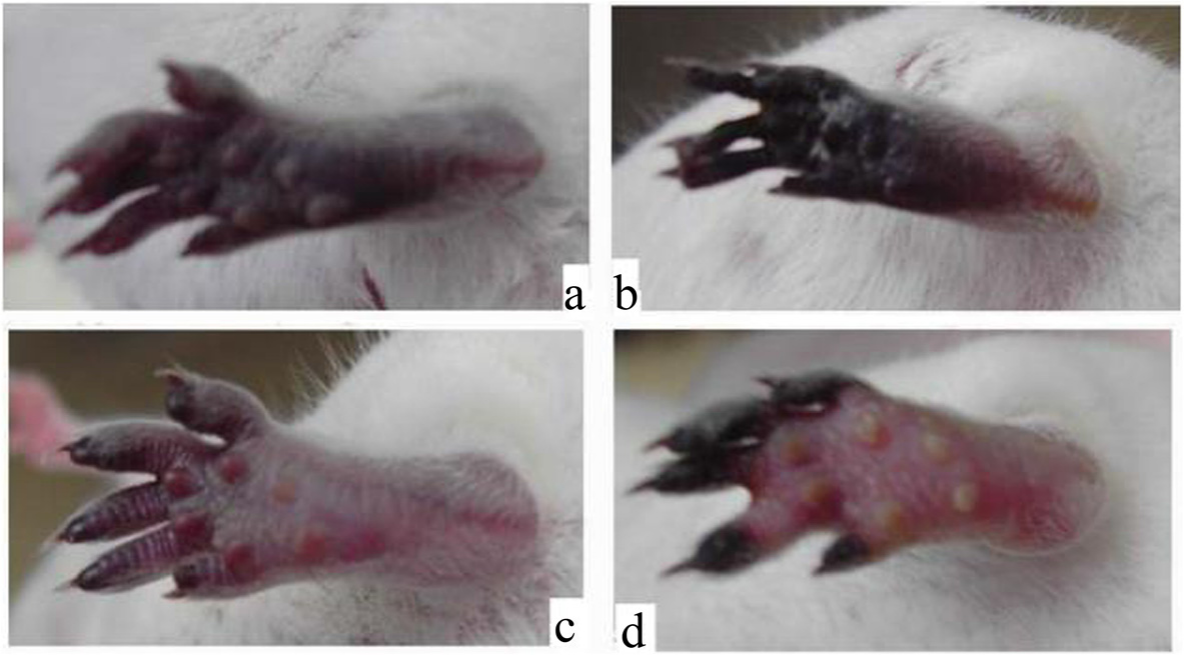
The effects of post-envenomation treatment with tirofiban, and clopidogrel or heparin. **a** One day after tirofiban + clopidogrel treatment. **b** Two days after tirofiban + clopidogrel treatment. **c** One day after tirofiban + heparin treatment. **d** Two days after tirofiban + heparin treatment

The difference in outcome between the groups is shown in Fig. 4 and Fig. 5. The best result occurred in the tirofiban + aspirin group: the ischemia was completely prevented. Necrosis was noted at the tip of the digits in the tirofiban + heparin group. The tip of the digits and some plantar necrosis was noted in the single-use tirofiban group. Severe plantar necrosis was noted in the tirofiban + clopidogrel group. The outcome in all treated groups was better than that in the control group.

### Single use of clopidogrel and heparin

**Fig. 6:**
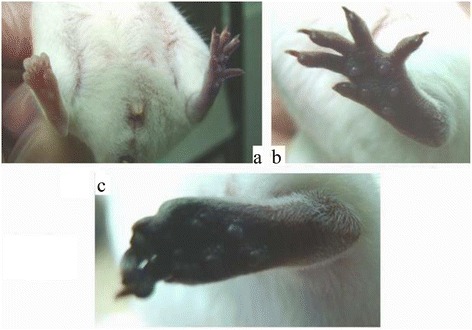
The effects of post-envenomation treatment with a single dose of clopidogrel. **a** clopidogrel (1 mg/BW 25 g) 8 h later. **b** clopidogrel (1 mg/BW 25 g) 1 day later. **c** clopidogrel (1 mg/BW 25 g) 2 days later

**Fig. 7:**
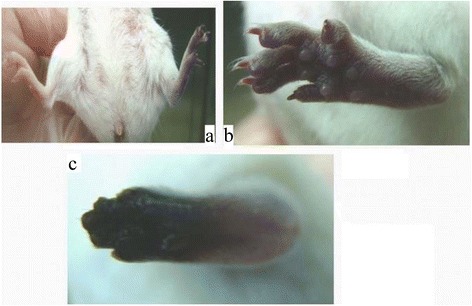
The effects of post-envenomation treatment with a single dose of heparin. **a** heparin(5U/BW 25 g) 8 h later. **b** heparin(5U/BW 25 g) 1 day later. **c** heparin(5U/BW 25 g) 2 days later

The clopidogrel group was slightly better than the heparin group, but the single-use clopidogrel group and single-use heparin group had virtually no effect on the prevention of tissue necrosis and gangrene (Fig. 6 and Fig. 7). Necrosis was noted and distal digits amputated in the heparin group. The clopidogrel group showed tissue necrosis, but amputation was not necessary. The difference between the treatment group and control group was not so clear. The tirofiban group showed a better outcome than that in the heparin group and clopidogrel group at identical observation points.

## Discussion

Formosan Russell’s viper is the sixth most frequent cause of poisonous snakebites in Taiwan [11]. As a result of urbanization, Formosan Russell’s vipers have gradually moved towards undeveloped regions. The time interval between snakebite and administration of antivenom is the major factor of successful treatment [12, 13]. Patients bitten by Formosan Russell’s viper (particularly in southern and eastern Taiwan) are usually far away from hospitals that have this antivenom, so replacing the antivenom with another medical treatment is crucial. Coagulopathy is the major effect of RVV envenomation [3, 14]. We tested various antiplatelets and anticoagulants to control the effect of coagulopathy to improve the outcome of RVV envenomation.

The component RVV-X in RVV acts on factor X to increase the amount of thrombin. An increased concentration of thrombin activates platelets and enhances clot formation. Platelet-rich clots are resistant to fibrinolysis [15]. Antiplatelet agents may help to prevent further activation and aggregation of platelets, and reduce the ratio of platelets compared with other components in clots.

Aspirin alone at a high dose showed obvious protection from venom-induced ischemia and necrosis of tissue (Fig. 3). The effect of aspirin was dose-dependent, but the adverse effects of high-dose aspirin are internal bleeding and bleeding tendency, and the dose is relatively high in humans. The recommended maximum dose of aspirin for anti-inflammatory purposes is 4500 mg/day [16]. Aspirin is relatively inexpensive and readily available in pharmacies. It has been used for decades as an analgesic and for the prevention of thrombosis. Aspirin can be used via the oral route and is very convenient for using outside the hospital setting. For reducing aspirin dosage, we tried to combine it with other drugs.

From the results of combined use of dual antiplatelet agents, tirofiban + aspirin produced a better outcome than that in the other groups (Fig. 4 and Table 1). All mice feet fully protected without the use of antivenom. The aspirin dose was reduced from 40 mg/kg to 10 mg/kg, an acceptably low analgesic dose [16]. The effect of tirofiban + heparinon outcome was moderate. However, the recovery in mice feet was partial, and the toe tips were necrotized, but outcome was better than in the single-use tirofiban group. The tirofiban + clopidogrel group showed no difference from the control group. Tirofiban + aspirin could completely reverse the thrombotic effects induced by the venom toxin of Formosan Russell’s viper without the use of antivenom.

Aspirin is well known for its antiplatelet properties, and is used to treat ischemic stroke, angina and acute myocardial infarction [17]. Aspirin irreversibly inactivates cyclooxygenase and then blocks thromboxane formation [18]. Thromboxane acts as a strong signal to amplify platelet activation by inducing irreversible platelet aggregation [19]. Thromboxane inhibits the activity of adenyl cyclase, leading to intraplatelet activation signals [20]. A decreased level of thromboxane inhibits the aggregation of platelets at injured endothelium, and blocks clot formation [21].

Heparin is used in stroke [22], acute coronary syndrome [23], deep-vein thrombosis [24–27], pulmonary embolism [24–27] and in cardiopulmonary bypass due to its anticoagulation effects. Heparin combined with antithrombin then inactivates thrombin, but has no effects on thrombin already adhered to fibrin [28]. The heparin–antithrombin complex can inhibit activation of factor X with high specificity [29], but heparin has little effect on already formed clots [30].

ADP binds to the Gq-protein-linked P2Y1 receptor on platelets, causing a change in cell shape, calcium mobilization, and initiation of reversible aggregation [31]. ADP also binds to the Gi-linked P2Y12 receptor on platelets to amplify aggregation via adenylylcyclase-mediated production of cyclic AMP [32]. Several studies reported the important role of the P2Y12 receptor in platelet thrombus formation and stabilization of collagen-coated surfaces under flow conditions [33–35]. The resulting platelet activation triggers a conformational change in glycoprotein IIb/IIIa receptors, which increases their affinity for fibrinogen. Clopidogrel acts on the ADP receptor on the platelet surface and inhibits platelet activation [36, 37], but has no effects on collagen-mediated activation and aggregation of platelets. We hypothesize that the observed endothelial damages were results of the exposure of collagen fibers to zinc metalloproteinase haemorrhagins that are present in RVV. This hypothesis may explain the lack of platelet inhibition by clopidogrel in this study.

Tirofiban is non-peptide small molecule that acts as an inhibitor of the glycoprotein IIb/IIIa receptor complex on the surface of platelets. It is used with aspirin or heparin in the treatment of angina and non-Q myocardial infarction [23]. The surface glycoproteins of platelets can combine with exposed collagen fibers in the vessel wall. Inhibition of platelet adherence to collagen fibers can prevent platelets from activation and aggregation, thereby stopping clot formation.

Aspirin inhibits the aggregation of platelets and stabilizes platelets, preventing activation then blocking clot formation. The inhibition is irreversible until new platelets are produced. Combined use of a glycoprotein IIb/IIIa inhibitor and aspirin stabilized platelets from activation and stopped clot formation. Platelet phospholipids help to activate factor X in the intrinsic pathway, and the formation of thrombin from prothrombin in extrinsic and intrinsic pathways. Platelet stabilization therefore prevents prothrombin being formed from thrombin. McFarlane was the first to identify activation off actor X in RVV [38–40]. RVV-X (the factor X-activating enzyme from RVV) has been well characterized as a proteinase [41, 42]. Irrespective of the rapidity of treatment for RVV envenomation, the RVV-X in venom will act as factor X before treatment to start to coagulation cascade. RVV-X acts before heparin in almost all conditions of envenoming, and coagulation started immediately after envenoming, Heparin works as an anticoagulant to prevent further clotting but cannot inhibit the action of platelets, and has no effect on clotting that has already occurred. Heparin therefore cannot reverse the effects of RVV for tissue ischemia and gangrene. Outcome in the aspirin plus tirofiban group with respect to prevention of tissue ischemia and gangrene was better than that in the heparin group. This indicated that the activation and aggregation of platelets was the major reason for thrombosis induced by RVV.

## Conclusion

Polyvalent antivenom is the “gold-standard” treatment for envenomation of Formosan Russell’s viper. However, antivenom just neutralizes the snake toxin to block the subsequent coagulation cascade, but there is no evidence that it affects already formed fibrin (or even thrombosis). The reversal and prevention of toxin-induced DIC and renal failure of antivenom is effective only if administered early. From the present study, aspirin + tirofiban showed an excellent outcome without the need for antivenom. We suggest the use of aspirin plus tirofiban should be used for envenomation by Russell’s viper irrespective of whether specific antivenom is available or not. Antivenom is reserved and prescribed only in specific hospitals in Taiwan, but aspirin and tirofiban are available in most hospitals. The accessibility of aspirin is much higher than antivenom, particularly for mountaineers and villagers far from such specialist hospitals. For victims of snakebites far away from such hospitals, we tried to afford effective and safe first-aid to prevent the adverse effects of RVV.
